# The effects of barbell resistance exercise on information processing speed and conflict-related ERP in older adults: a crossover randomized controlled trial

**DOI:** 10.1038/s41598-021-88634-5

**Published:** 2021-04-28

**Authors:** Ting-Yu Lin, Shu-Shih Hsieh, Ting-Yu Chueh, Chung-Ju Huang, Tsung-Min Hung

**Affiliations:** 1 Department of Physical Education, National Taiwan Normal University, Taipei, Taiwan, ROC; 2 Department of Psychology, Northeastern University, Boston, MA USA; 3 Graduate Institute of Sport Pedagogy, University of Taipei, Taipei, Taiwan, ROC; 4 Institute for Research Excellence in Learning Science, National Taiwan Normal University, Taipei, Taiwan, ROC

**Keywords:** Psychology, Human behaviour, Cognitive control, Perception, Attention, Neuroscience, Cognitive ageing, Cognitive neuroscience

## Abstract

It is difficult to draw conclusions about the effect of resistance exercises on information processing speed and inhibitory control from previous studies due to possible underestimations of maximal strength and the lack of information on the intervention programs. To address this issue, a familiarization of resistance exercise was introduced before the strength test, and the repetition-to-fatigue method was used to calculate the 1RM (one repetition max). A two-arm RCT was conducted to evaluate the cognitive effect of resistance exercise. Male adults aged 50–65 years old performed a single bout of multiple joint, structural barbell resistance exercises (back squat, press, and deadlift) with 75% 1RM * 5 repetitions * 3 sets with 2–3 min rest between sets and exercises or a stretching exercise session (active-control intervention). This type of resistance exercise improved the information processing speed measured by Stroop task reaction time (*t*(23) =  − 2.313, *p* = .030, *M* =  − 16 ms, 95% CI [− 30, − 2]) and decreased the conflict-related neural activity measured by event-related potential N2b in both congruent (*t*(20) = 2.674, *p* = .015, *M* = 2.290 μv, 95% CI [0.504, 4.075]) and incongruent (*t*(20) = 2.851, *p* = .018, *M* = 2.291 μv, 95% CI [0.439, 4.142]) conditions. Resistance exercise significantly improved information processing speed and decrease conflict-related neural activity, but did not change inhibitory control in older adults compared to active control.

*Trial registration*: NCT04534374 (01/09/2020).

## Introduction

Information processing speed and inhibitory control are two critical factors in daily life and higher-order cognition^1,2^. Reaction time, a measure of information processing speed, is an indicator of the brain’s information processing ability^3^. Choice reaction time latencies have been estimated to increase by 1 ms/year in males after the age 20^4^, and an increase of mean choice reaction time is associated with a higher risk of cardiovascular, coronary heart, and respiratory disease mortalities after adjusting for sex, social-economic status and smoking^5^. Because slower information processing speed is a risk factor for mortality, reducing this decline is critical. Inhibitory control is one of three major components of executive function, which refers to a family of top-down mental processes, also called executive control or cognitive control. Inhibitory control makes it possible for us to change old habits of thought or action and prevent us from being controlled by impulses^1^. Inhibitory control, like other executive functions, declines during normal aging^6,7^ and disproportionately so if general health is compromised^1^. For instance, performance differences between younger and older participants are greater for tasks requiring a high-level inhibition compared to lower-demands condition^8^.

The Stroop test provides a measure of both inhibitory control and information processing speed, and the analysis of event-related potentials (ERP) recorded during this test also provides information about the underlying neuro-electrophysiologic mechanisms involved. ERPs' high temporal resolution allows for the investigation of the ordering and timing of different mental processes^9^. This, in turn, allows for a description of the stages of information processes and their durations^10^. N2b (anterior N2) has its largest amplitude in the frontocentral lobe^11^. Originating in the Anterior Cingulate Cortex (ACC), it is associated with conflict monitoring neural activity^12–14^, and is activated when multiple responses compete, a feature of the Stroop task^15–17^. The N2b amplitude is positively correlated with reaction time^14^ and it can be used to provide information about the mechanisms involved in determining thereof.

Resistance exercise (RE) has been shown to improve not only neuromuscular function but also executive function^18^. To our knowledge, there are 11 previous studies which have investigated the effects of acute RE on the performance of the Stroop task^19–29^. The type of RE is critical when constructing a RE program to maximize both cognitive and physical performance in the long-term. It has been proposed that multiple-joint, free-weight, structural (the force vectors directed primarily through the spine and hip^30^) resistance exercises will have greater effects relative to single-joint, machine-based, non-structural exercise on functional movement^31^, bone mineral density^32,33^, the release of neurotrophic factor^34–37^, and degree of muscle activations^38,39^. However, these proposals remain untested in previous studies^19–29^. It is also difficult to determine optimal RE protocols from previous studies primarily because of the possibility of underestimation of maximal strength, or unreported training parameters. The studies with unreported fundamental training parameters and training protocol with the risk of underestimation of maximal strength were summarized in Table 1. For comparison, various predicted equations of repetition max have been identified. Besides, only one of the 11 studies mentioned applied an active-control intervention^28^, and none applied intention-to-treat analysis, or familiarization before the strength tests. Thus, these studies may have over-estimated the effects of resistance exercise while participants’ maximal strength may have been underestimated. Therefore, the present study focuses on addressing the limitations of previous work by utilizing (1) a two-arm, crossover RCT design (2) a RE protocol that has been shown to be effective for improving muscular strength and relevant connective tissues (3) a familiarization with the strength test (4) an active-control physical test (flexibility test) and intervention (stretching, SE) (5) strict intention-to-treat analysis^40^ for behavioral data and (6) analysis of ERP data. We hypothesized that this type of acute RE would produce (1) improvements in information processing speed (2) improvements in inhibitory control (3) smaller N2b amplitude.

**Table 1:** Prediction Equations for repetition max (RM) and reports from cited studies.

From LeSuer, et al.^52^				
Author(s)	Equation	Value for comparisons (% 1RM)		
		5RM	9RM	10RM
Wathan	$1\mathrm{RM}=100\times \mathrm{weight}\div\left(48.8+53.8{e}^{-0.075\times reps}\right)$	86	76	74
Brzycki	$1\mathrm{RM}=100\times \mathrm{weight}\div\left(102.78-2.78\times \mathrm{reps}\right)$	89	87	75
Lander	$1\mathrm{RM}=100\times \mathrm{weight}\div\left(101.3-2.67123\times \mathrm{reps}\right)$	88	77	74
Epley	$1\mathrm{RM}=1\div\left(1+0.0333\times \mathrm{reps}\right)\times \mathrm{weight}$	86	77	75
Lombardi	$1\mathrm{RM}={\mathrm{weight}\times \mathrm{reps}}^{0.1}$	85	80	79
Mayhew et al	$1\mathrm{RM}=100\times \mathrm{weight}\div\left(52.2+41.9{e}^{-0.055\times reps}\right)$	84	78	76
O'Conner et al	$1\mathrm{RM}=\mathrm{weight}\times \left(1+0.025\times \mathrm{reps}\right)$	89	82	80
Reports from cited studies				

Author	Description			
Brush, et al.^23^	10 repetitions with 100% 10-RM for 3 sets			
Engeroff et al.^27^	5 repetitions with 90% 1RM for 5 sets			
Tsukamoto et al.^20^	10 repetitions with 80% 1RM for 6 sets			
Sardeli et al.^24^	9.6 (mean) repetitions with 80% 1RM for 4 sets			
Chang and Etnier^21^	Did not report rest intervals			
Chang et al.^22^	Did not report repetitions			
Harveson et al.^29^	Did not report intensity and rest intervals			

Note that the formulas are used to predict how many repetitions a participant can complete at a given weight for a single set.

## Method

### Participants

This study recruited 28 older men. Study selection criteria were: (1) male adults aged 50–65 years old; (2) free from dementia (Mini-Mental State Examination, MMSE ≥ 24^41^); (3) free from any medical condition listed on Physical Activity Readiness Questionnaire, PARQ^42^ (4) free from depression (Beck Depression Inventory-II, BDI-II score ≤ 13^43^); (5) free from any diagnosed cardiovascular, neurological, and other chronic diseases (6) exercise ≥ 150 min/week (7) normal or corrected-to-normal vision (8) Right-handed.

### Demographic and anthropometric measures

The health status of participants was assessed with reference to the PARQ. Dementia, depression, handedness, and physical activity were assessed against the MMSE, the BDI-II, the Edinburgh inventory^44^, and the International Physical Activity Questionnaire (IPAQ)^45^, respectively. Body mass index (BMI) was calculated as weight (kg)/height^2^ (m^2^). Economic status was assessed by self-report (measured on a 5-point scale with ‘5′ representing the highest income). The investigation was approved by the Institutional Review Board of the National Taiwan Normal University (NTNU). All data were collected within the NTNU gymnasium between August 2017 and August 2019. All experiments were performed in accordance with relevant guidelines and regulations.

### Experimental design

A crossover design was used because of its efficiency and the low probability that the known disadvantages of this design, such as carryover effects and instability of participants' conditions^46,47^, would occur in this study given adequate washout and the control of differences in circadian rhythm. After participants provided informed consent, they then completed the questionnaires, height, and weight measurements, and a familiarization of the resistance exercises on the first day.

**Figure 1:**
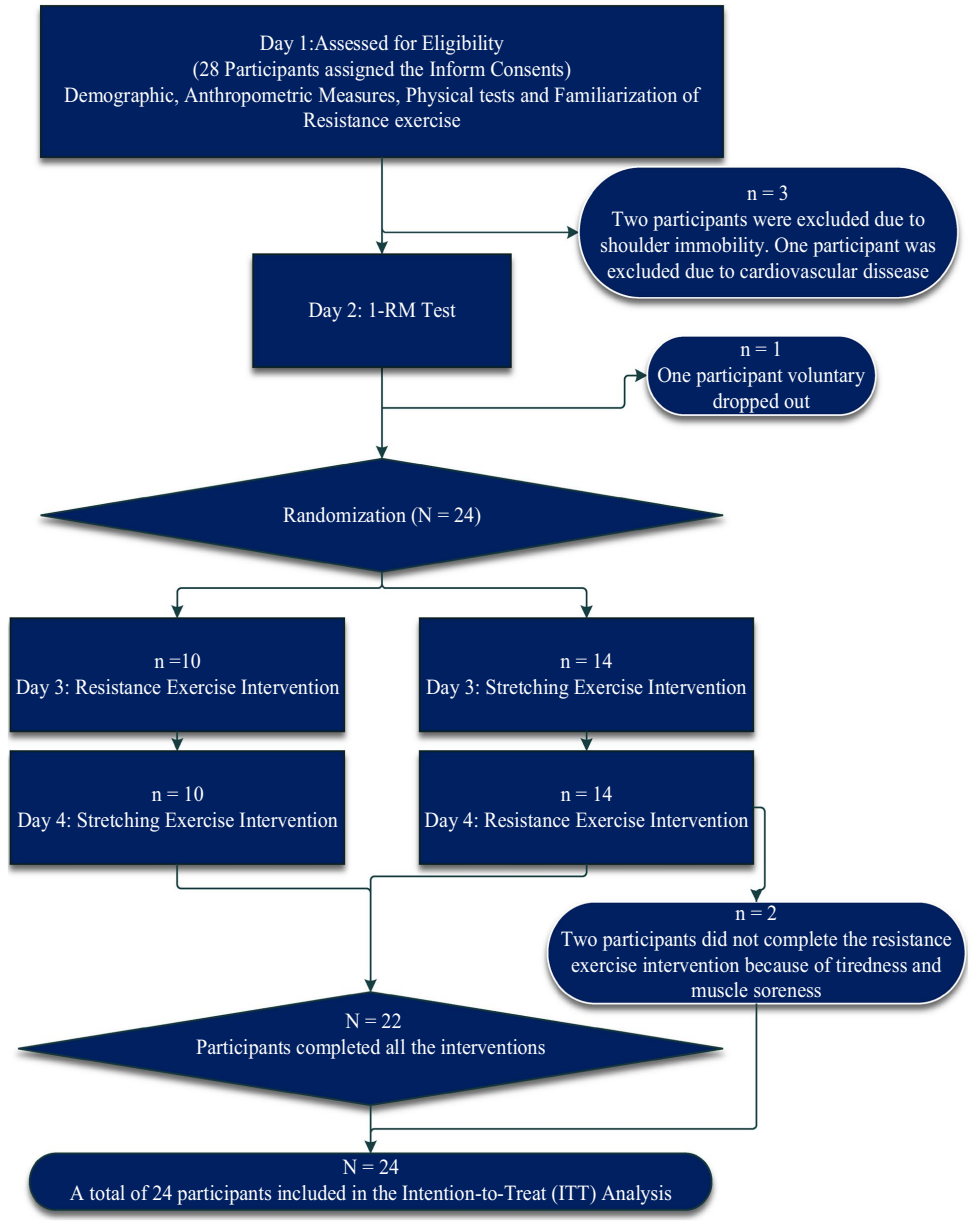
CONSORT diagram of participant flow.

On the second visit, participants completed the rep-to-fatigue 1-RM test for the barbell back squat, press, and deadlift exercises. All participants were randomly assigned to two different sequences. During days 3 and 4, participants completed the modified Stroop color-word test 5 min before and 10 min after the exercise interventions. The minimum interval between each visit was 96 h. Participants were required to visit the lab at a similar time of the day in all four visits to control for effects of variations in circadian rhythm on muscle strength and cognitive performance. Participants were asked to refrain from caffeine and alcohol for 12 and 48 h, respectively, and finish the last meal at least 2 h before the visit. They were also asked to avoid vigorous exercise for 48 h. All procedures were conducted one participant at a time supervised by TYL. Figure 1 presents the CONSORT flow diagram for crossover trials^47^. The participants and public were not involved in the research. Dissemination of results to participants will be done within 1 year after the trial is closed via an electronic document.

### Strength testing

One of the authors with about 5 years of experience in coaching (TYL, NSCA-CSCS & CSPS) instructed participants in barbell back squat, press and deadlift during day 1 of the familiarization session^30^. The instruction was in accordance with *Starting Strength 3*^*rd*^^48–50^ with two modifications:

Squat: both ‘thumb-around’ and ‘thumb-up’ grips were permitted according to which position felt most comfortable to participants.

Press: For the sake of simplicity, the static press was instructed without including a leaning back of the trunk or dynamic movement of the hips.

On day 2, after following the NSCA’s warm-up protocol for maximal strength test^30^, the repetition-to-fatigue method (Wathen’s equation^51^ was chosen because it was the most accurate one reviewed by Leseur et a.l^52^) was used to estimate the 1-RM because a direct measure of this may have been unsuitable for those not accustomed to resistance exercise^30^. This equation has been shown to have low errors of measurement and high Pearson correlations coefficient between predicted and directly measured 1-RM^52^ (differences: 0.8%, 0.02%, and 9% for bench press, squat, and deadlift, respectively; correlations for the same three exercises were 0.992, 0.969, and 0.965, respectively). The target weight in the testing was that the participants could perform 3–10 repetitions. This is because NSCA does not recommend performing testing with weight over 3RM^30^ and ≤ 10 repetitions to fatigue is better for estimating 1RM^53,54^. For instance, if a participant can perform an 80 kg squat for 7 repetitions at most, the $1\mathrm{RM}=100\times 80\mathrm{ kg}\div\left(48.8+53.8{e}^{-0.075\times reps}\right)= 99\mathrm{ kg}.$ Strength for each exercise movement, and the sum of all movements, was measured in terms of absolute strength (weight lifted), relative strength (weight lifted/body weight), and allometric strength (weight lifted/body weight^0.66^).

### Flexibility testing

Flexibility tests were performed, although not analyzed, to further make participants in the stretching exercise condition believe this is also an experimental rather than an active control condition. Measures taken were the sit-and-reach^55^ and shoulder mobility tests^56^.

### Modified Stoop color-word test

Information processing speed and cognitive inhibition were measured by participation in the modified Stroop color-word test^57,58^. STIM 2.0 software (Neuroscan Ltd, El Paso, TX, USA) was used to create and present the task. The task involved both congruent and incongruent conditions. In the congruent condition, one of three color words, written in Chinese characters were presented in the color that they referred to. For example, the character  , which means “red”, was written in red color. The other two colors used were yellow and green. The incongruent condition presented characters in one of the two non-congruent colors with equal frequency. Thus, in the incongruent condition, the character  (which means yellow) was written one half of the time in red and one half of the time in green.

The stimuli, 2 × 2 cm in size, were presented in the center of a 17-inch monitor placed 75 cm in front of participants at a visual angle of 2 degrees. Participants were asked to respond as accurately and as quickly as possible to the color, but not the meaning of the character, by pressing “G” for red, “H” for yellow, and “J” for green on the keyboard. The time allowed for presenting the stimuli, reacting to it, and the inter-trial interval were 500 ms, 1500 ms, and 500 ms, respectively. Trials with no response, or a reaction time shorter than 200 ms, or longer than 1500 ms, were marked as incorrect. The ratio of congruent vs incongruent trials was 7:3. There were a total of six blocks with each block consisting of 60 trials, i.e. a total of 360 trials. Before the formal experimental procedures, participants were allowed to practice on a block of 20 trials. If the accuracy of this practice block ≥ 85%, all subsequent trials were recorded. When accuracy was below this criterion, participants were allowed a second practice block. If a participant also failed to reach the required accuracy in this second block, they were excluded from the study. The entire task (including practice trials) required about 25 min.

Information processing speed was measured as the mean reaction time of congruent trials, and cognitive inhibition was calculated as the time difference between the mean reaction time of congruent and incongruent trials (interference score, IF score). Response accuracy was also calculated to see if there were significant differences in the speed-accuracy trade-off between the resistance exercise and active control interventions.

### Resistance exercise intervention

**Figure 2:**
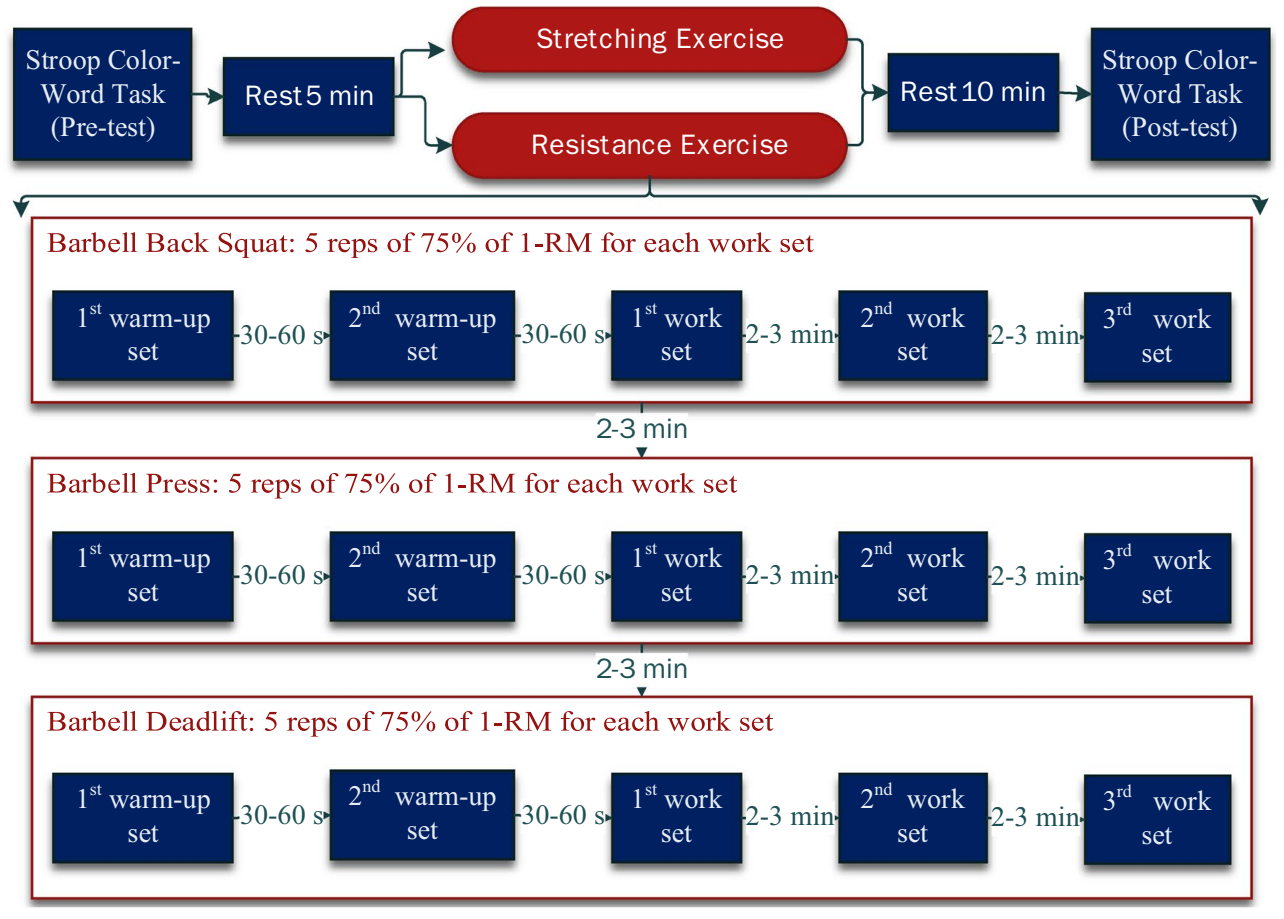
The procedure of exercise interventions.

On the resistance exercise day, participants performed barbell back squat, press, and deadlift for three sets of five repetitions with the weight closest to 75% of their estimated 1-RM^59,60^ for all three movements mentioned previously. The participants were instructed to execute the movement with moderate speed (2 s eccentric, 2 s concentric, no pause in between). The rest period between sets and exercise movements was 2 to 3 min, in line with the NSCA’s recommendation for strength training^30^, see Fig. 2. The Rating of Perceived Exertion (RPE) scale (score: 6–20)^61^ was used to assess perceived exercise intensities. The RPE scores were collected immediately after each working set of each exercise type, a total of 9 RPE scores were reported during the exercise session (see Supplementary Table S1 online).

### Active-control exercise intervention (stretching)

On the control intervention day, participants completed a passive stretching exercise session for a similar duration of time as the resistance exercise (≈ 30 min). Participants performed 15–20 stretching movements twice, holding each for 30 s as recommended by ACSM^55^.

### Determination of sample size

The number of participants required was calculated by the average effect size (partial η^2^ $=\left(0.32+0.26\right)\div 2=0.29$) reported in a previous study^23^. The reason this study was chosen is that it was the only published paper that reported Stroop interference scores. The partial η^2^ was transformed to Cohen’s d ($2\times \surd ({0.29}^{2}\div\left(1-{0.29}^{2}\right))\approx 0.61$) then a sample size of 23 was computed according to the equation $\mathrm{n}=\frac{8}{{d}^{2}}+2$^62^ sufficient to be able to achieve a power estimate of 0.8 assuming α = 0.05 in a single sample difference from constant *t* test, the approach recommended for crossover trials^47^. A total of 28 participants were recruited to ensure that sample size would still be adequate assuming a drop out rate before randomization of up to 15%’.

### Event-related potential (ERP)

#### Electroencephalographic (EEG) recording

The 32 channels elastic electrode cap (Quick-Cap, Compumedics Neuroscan, Inc., Charlotte, NC, USA) was used according to the modified International 10–20 System to record electroencephalographic activity (EEG). Four electrodes were placed above and below the left orbit and outer canthus of each eye to record electrooculographic activity (EOG). Two vertical EOG electrodes and two horizontal electrodes were combined into two external channels (VEOG and HEOG). EEG data was re-referenced to the average of two mastoids (M1 and M2). All electrodes kept impedances < 10 kΩ before data collecting. Neuroscan SynAmps2 amplifier was used to continuous data recording with 1000 Hz sampling rate, DC- to 200-Hz online filter, 60-Hz online notch filter, and AFZ electrode as the ground.

#### EEG processing and averaging

1. Converting .cnt file (collected by Neuroscan 4.5) to .set file using Matlab 2019b and its add-on EEGlab version 14^63^.
2. Removing data collected more than 2 s outside of triggers and appending.
3. Bandpass (IIR, 0.1–30 Hz half amplitude cut off, 12 dB/oct roll-off for both high and low pass) by the EEGlab’s plugin ERPlab version 7^64^.
4. Rejecting and interpolating bad channels (measures used: normal distribution and kurtosis, criteria: ± 5 SD, interpolation: spherical) by EEGlab.
5. Epoching (correct trials, − 200–1000 ms to the start of the stimuli) and rejecting bad epochs (measures used: normal distribution and kurtosis, criteria: ± 5 SD)^65^ by EEGlab.
6. Relocating external channels (VEOG and HEOG) and run ICA (runica, by EEGlab).
7. Selecting and removing ICA components associated with the eye-blink artifact with Icaeyeblinkmetrics version 3.2 electrodes used: VEOG and FP1^66^ after converting the epoch file to a continuous one by ERPlab.
8. Re-epoching (baseline correction: − 200–0 ms) and trial rejection using step function channel: all internal channels, threshold: 100 μv, time window: − 200–1000 ms, window size: 200 ms, window step: 50 ms, ERPlab^9^.
9. Computing average ERP and drawing the topography plot for congruent and incongruent conditions by ERPlab.

Among the 22 participants who completed all exercise interventions, ERP data from one of the participants were rejected because it was unable to undergo ICA blink rejection due to bad channels. A total of 21 participants’ data was included in the ERP analysis.

#### Quantifying ERP

The N2b component was defined as the local peak over 25 sampling points 180–325 ms^11^ after stimuli calculated by ERPlab, ERP measurement tool^9^. The local peak amplitude was computed by averaging the 25 sampling points. To decrease the level in statistical analysis^67^, nine frontocentral electrodes were average (F3, FZ, F4, FC3, FCZ, FC4, C3, CZ, and C4)^11^.

### Data analysis

SPSS 23 software was used to perform the statistical analysis. All randomized participants were included in the analysis (intention-to-treat analysis, ITT, see Fig. 1) for primary (behavioral) data. The last-observation-carried-forward (LOCF) method was used to process the missing outcome. One sample difference from constant *t* tests ((post-RE − pre-RE) − (post-SE − pre-SE)) were performed for reaction speed and accuracy data under congruent and incongruent conditions, as well as N2b local peak amplitude (post-test minus pre-test). The α was set at 0.05 and the effect size was reported in contrast confidence intervals (95% CI) along with *p* values^40,68,69^, which would allow future meta-analyses to input effect sizes directly. The confidence interval for each intervention, Cohen’s d, along with within-participants Pearson correlation coefficients, were reported to allow comparisons with previous studies and to calculate the required sample sizes for future studies^62^. The minimal detectable difference (MDD) was calculated from both baseline scores in order to make comparisons with intervention effects, to verify that changes occurring after experimental sessions were greater than outcomes’ normal variability. It was calculated as the one standard error of the measurement (1 SEM): SD $\times \sqrt{(1-r})$, where SD was the standard deviation of the change score (first pre-test − second pre-test) and r was the Pearson correlation coefficient^70,71^.

#### Controlled risk of bias and measurement variability

1. Bias arising from randomization process (allocation generation and concealment): Random numbers (1 and 2) were generated using Excel 2016 (allocation ratio: 1:1) and these were used to allocate participants to either resistance exercise followed by the active-control intervention or vice versa to participants (unrestricted randomization) after they had agreed to participate in this study to avoid allocation bias^72^.
2. Bias arising from missing data: Except for dropouts, there was no missing data relating to cognitive performance. However, a certain amount of ERP data loss due to noise was unavoidable.
3. Bias arising from outcome measurement: The computerized cognitive task and fully automatic data analysis of behavior and ERP analysis minimized bias in outcome measurement.
4. Blinding: Participants were blinded as to the treatment under investigation by use of an active-control physical test (flexibility test) and intervention (stretching).
5. Bias due to deviations from intended interventions: The washout period between the two interventions was at least 96 h to minimize any carry-over effects.
6. One of the authors (TYL) performed all physical examinations, exercise instructions, and intervention supervision individually with every participant to avoid inter-rater and inter-instructor variability. The same author also enrolled the participants, generated the random allocation sequence, and assigned the interventions.

### Ethical approval

This study was approved by the NTNU institutional review board (201807HM004).

## Result

### Completion rate, adverse events, demographic, anthropometric measures, and outcomes of strength and flexibility tests

28 participants were initially recruited, 24 of whom were included in the strict intention-to-treat analysis, and of these 15 participated in the active-control intervention before the resistance exercise intervention. Of the four participants who did not take part in any intervention, two were excluded because of shoulder immobility, one was excluded because of a cardiovascular issue, and the other voluntarily dropped out (Fig. 1). There was no severe adverse event. Two out of the remaining 24 participants did not complete the resistance exercise intervention because of tiredness and muscle soreness, giving an adherence rate of 92% (22/24). The interventions were delivered as planned for all 22 participants. The demographic, anthropometric measures, and outcomes of strength and flexibility tests are shown in Supplementary Table S1 online.

### Subjective exercise intensity

The RPE for each set of each exercise movement is shown in Supplementary Table S2 online. The average RPE was 13.6 ± 1.4.

### Behavioral outcome (primary outcome)

#### Stroop effect

Reaction times were shorter and accuracy was higher in congruent conditions compared to incongruent conditions in all pre-RE, post-RE, pre-SE, and post-SE sessions. All *p* values measured by paired t-test were ≤ 0.0014.

#### Information processing speed

All participants reached an ≥ 85% accuracy level after the two practice blocks. RE significantly improved reaction time, *t*(23) =  − 2.313, *p* = 0.030 M =  − 16 ms, 95% CI [− 30, − 2] (Table 2). The mean intervention effect was larger than the minimal detectable change which was 11 ms ($38.44 \times \sqrt{(1-.917}$).

**Table 2:** Results for effects of each intervention and within-participants comparisons.

Variable	Resistance exercise Mean (SE)	Stretching exercise Mean (SE)	Contrast Mean [95% CI; *p* value; Cohen’s d; within participant Pearson correlation coefficient (*p value)*]
Behavioral outcomes (n = 24)			
Reaction time (ms)	− 33 (9)	− 17 (6)	− 16 [− 30 to − 2; .030; − 0.47; .625 (.001)]
Interference score (ms)	5 (10)	0 (10)	6 [− 12 to 23; 515; 0.13; .612 (.001)]
ERP outcomes (n = 21)			
N2b local peak amplitude (μv)			
Congruent condition	2.210 (0.409)	− 0.080 (0.659)	2.290 [0.504 to 4.075; .015; 0.58; − .241 (.292)]
Incongruent condition	2.232 (0.381)	− 0.589 (0.695)	2.291 [0.439 to 4.142; .018; 0.56; − .302 (.183)]

#### Inhibitory control

There was no significant effect of RE on interference scores, *t*(23) = 0.661, *p* = 0.515, *M* = 6 ms, 95% CI [− 12, 23], see Table 2.

#### Accuracy

The analysis of response accuracy showed no significant effects of RE in either the congruent (*t*(23) = 0.131, *p* = 0.897, *M* = 0.03%, 95% CI [− 0.49, 0.56]) or incongruent conditions (*t*(23) =  − 0.525, *p* = 0.604, *M* =  − 0.4%, 95% CI [− 2.10, 1.25]).

### Event-related potentials (secondary outcome)

#### N2b local peak amplitude

**Figure 3:**
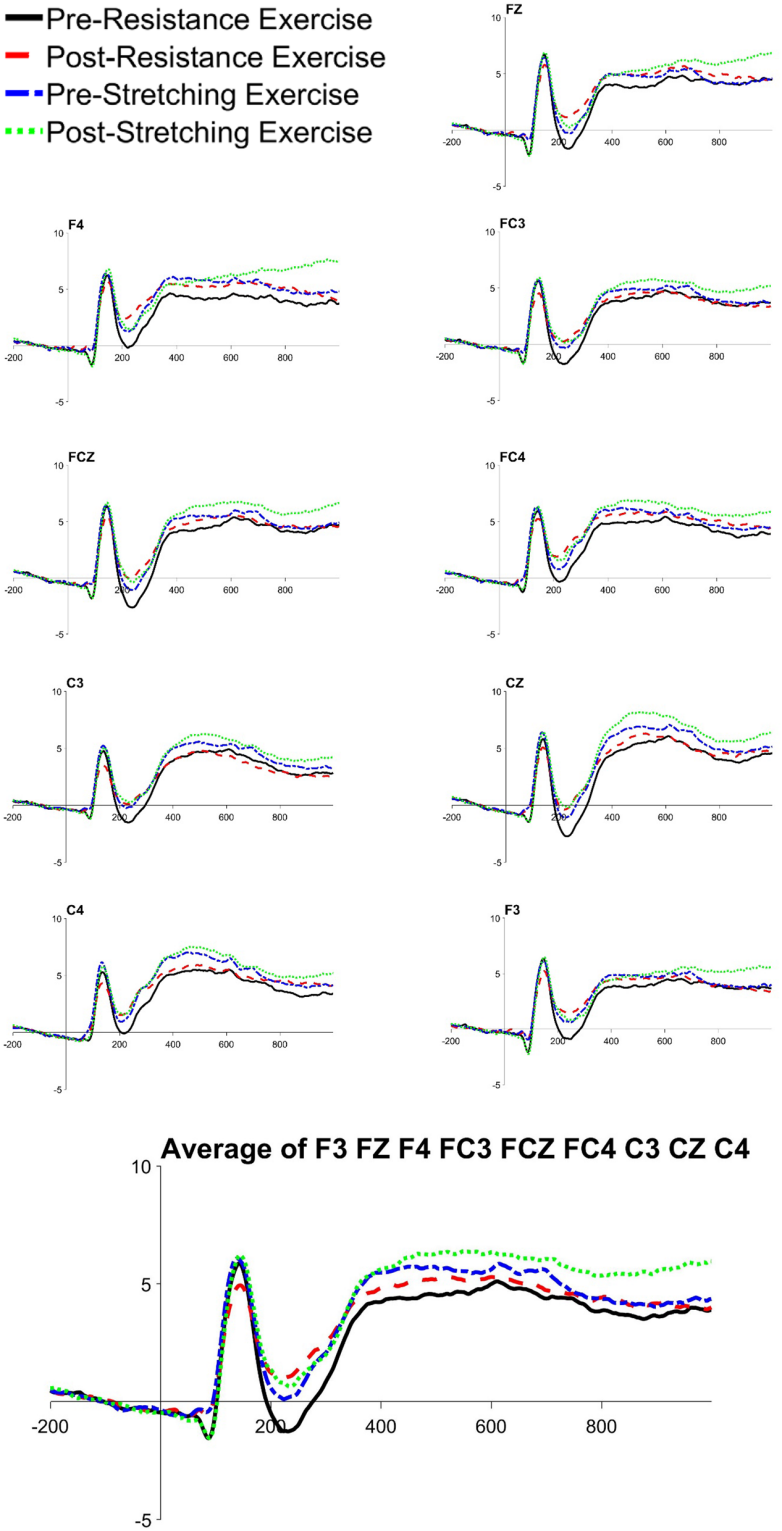
The grand averages of frontocentral electrodes in congruent condition. Drawn by ERPlab 7.0^64^.

**Figure 4:**
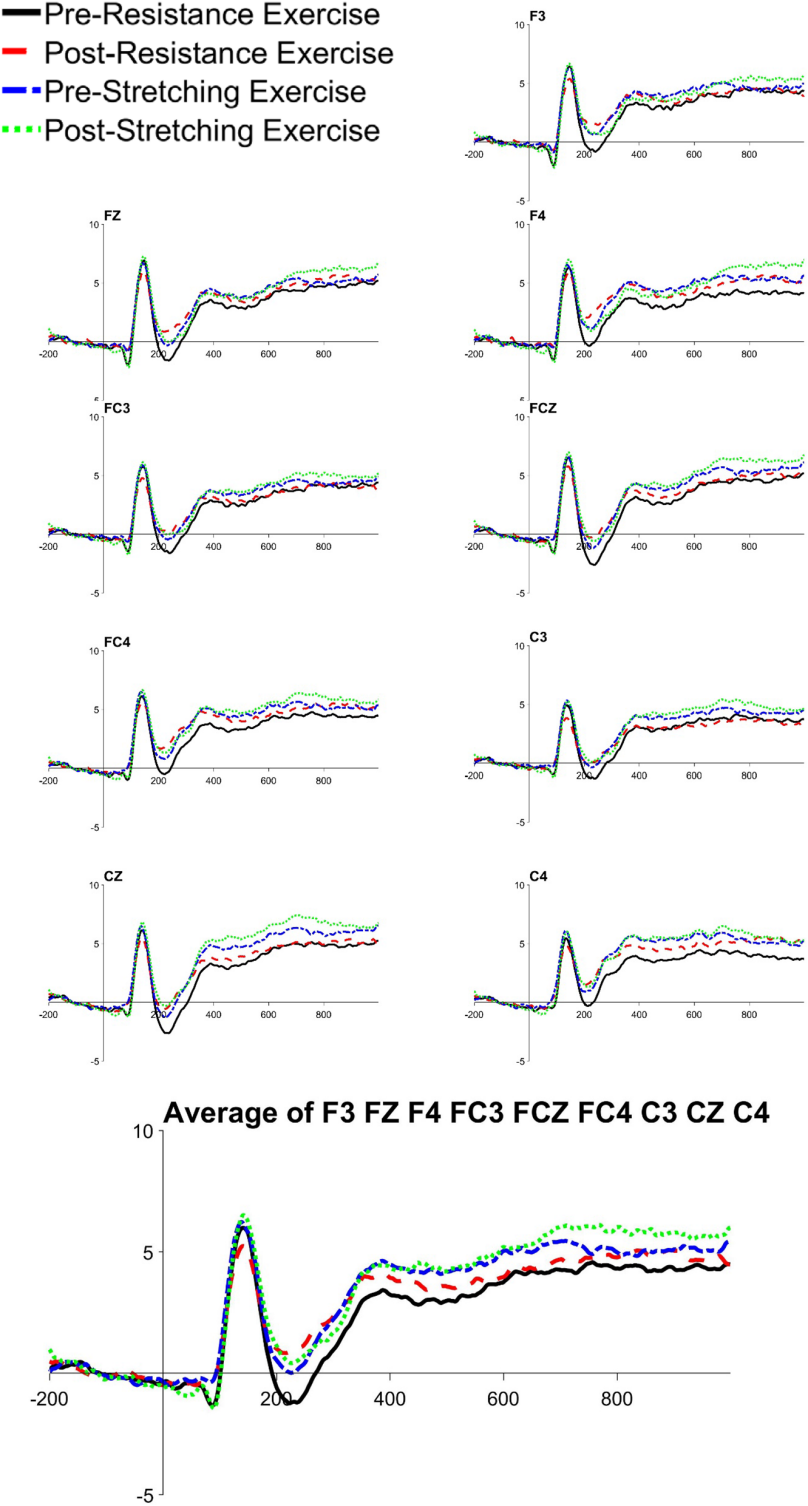
The grand averages of frontocentral electrodes in incongruent condition. Drawn by ERPlab 7.0^64^.

**Figure 5:**
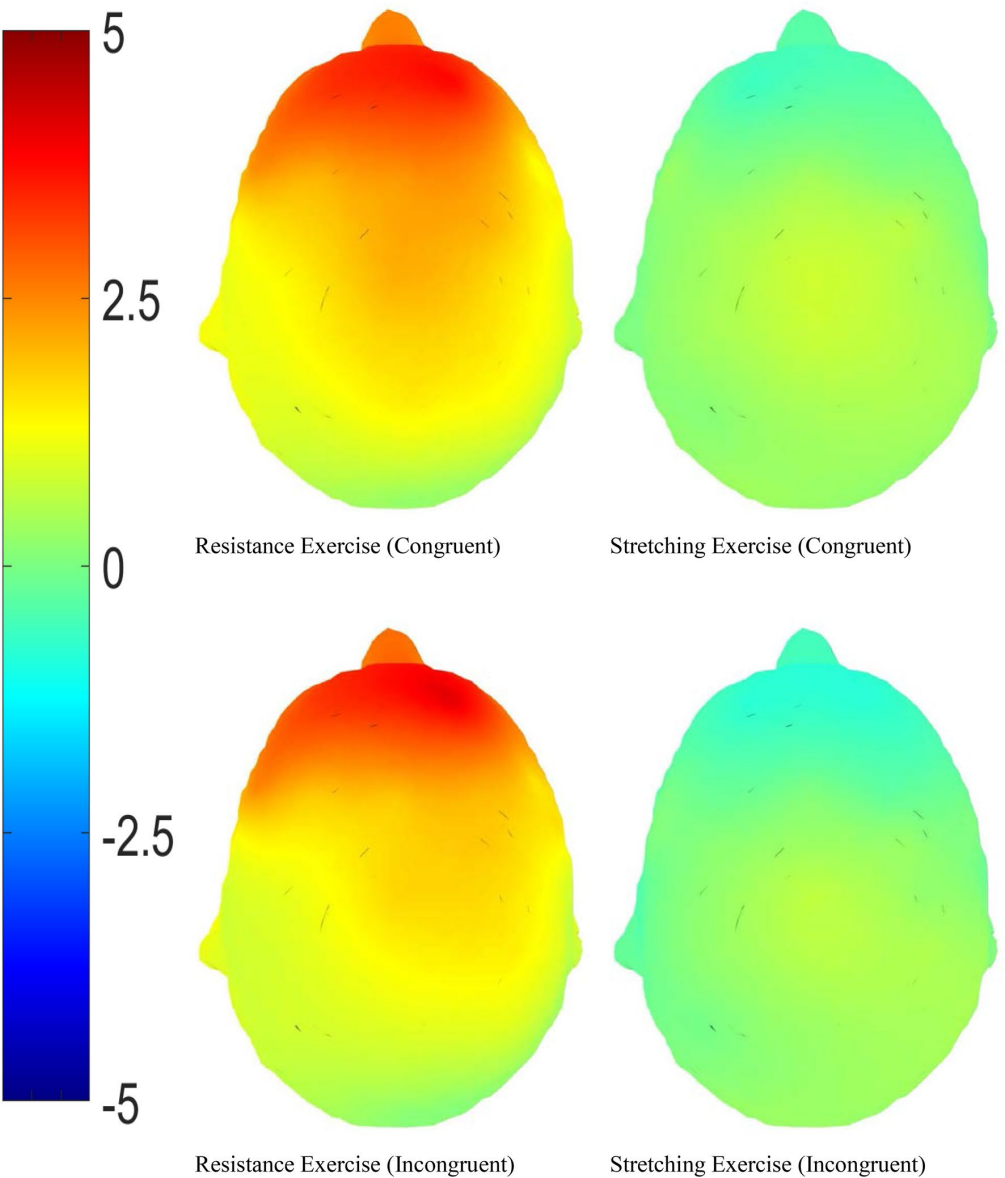
N2b Topographic Mapping (top view). Comparisons between changes (post-exercise − pre-exercise) of resistance exercise and stretching exercise in congruent and incongruent conditions. Mean amplitude between 180 and 325 ms post stimuli. Drawn by ERPlab 7.0^64^. (unit: μv).

There was a significant difference in N2b local peak amplitudes between congruent and incongruent conditions, *t*(20) = 3.242, *p* = 0.004, with amplitudes being larger in the incongruent condition. In addition, RE significantly reduced the N2b local peak amplitude in both the congruent (*t*(20) = 2.674, *p* = 0.015, *M* = 2.290 μv, 95% CI [0.504, 4.075]) and incongruent condition (*t*(20) = 2.851, *p* = 0.018, *M* = 2.291 μv, 95% CI [0.439, 4.142]), see Table 2, Figs. 3, 4 and 5.

## Discussion

Acute multiple joint, structural barbell exercises were found to significantly improve information processing speed and decrease conflict-related neural activity, but did not change inhibitory control in older adults. As expected, both the shortening of reaction time and the reduction of N2b amplitudes were greater in the RE condition compared to the active-control condition. The mean difference in reaction time between the two interventions was 16 ms (Table 2), the average change in processing speed typically seen in populations 15 years apart in age ($16\div 1.1$)^4^. As for inhibitory control, there was no significant difference in Stroop interference scores as a result of RE intervention.

### Comparison with previous studies

Published studies have reported an inconsistent effect of acute RE on information processing speed in the Stroop task. Results from five of 11 previous studies were excluded from the following discussion because two studies^19,27^ did not have a control group; one study^20^ used the reverse Stroop task (word-naming rather than color-naming); one^28^ did not report results of the comparison between RE and the control intervention (only the pre- vs post-test comparison); one^26^ asked participants to identify congruency between the word and its color rather than the color of the word per se. The Stroop test can be used in several ways to collect information processing speed (e.g. responding to the meaning of a word in black ink, naming the color of a dot, or determining the color of a color-unrelated word), while only those that present potentially incongruent information (e.g. the word ‘red’ written in green color) can be used to test inhibition.

Combining all non-interference/congruent measures from the remaining six studies, there were 19 comparisons of reaction speed between RE and control interventions. To prevent the underpowered studies from being inappropriately assessed as not showing benefit, both the direction of effects and statistical significance will be discussed^73^. Eleven of the 14 comparisons indicating that RE favored information processing speed reached statistical significance^21–25,29^, whereas only one in five comparisons favoring the control interventions was significant^23^. Although these studies used similar forms of Stroop tasks to measure cognitive performance, it is difficult to compare these results directly with the current study due to the methodological and reporting limitations included potential underestimation of maximal strength, unreported information related to the intervention program, lack of active control and ITT analysis. Among the six studies, only one reported Stroop interference scores^23^. There was a total of six comparisons of interference scores, all favored RE but only one reached significance (high intensity, 15 min post-exercise).

### Potential mechanisms

**Figure 6:**
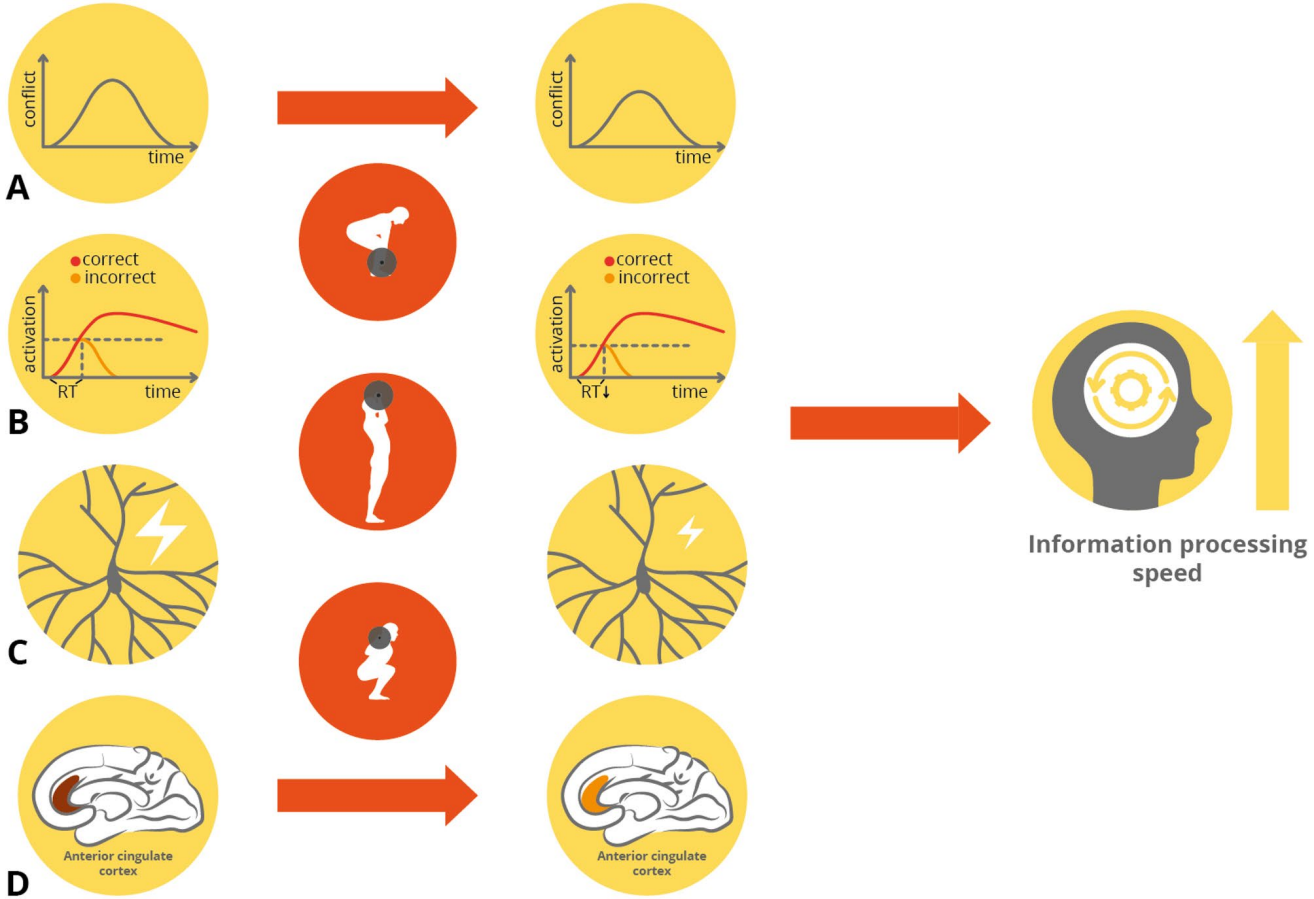
The potential mechanism underlying the improvement of information processing speed. (**A**) The conflict lessened as measured by the decreased N2b amplitude. (**B**) The decrease of conflict may attribute to the reduction of activation of incorrect along with a lower threshold for responses (dotted line). (**C**) and (**D**) The decrease of N2b amplitude reflects the lessened conflict in ACC.

The improvement in information processing speed may be attributed to reduced neural activation associated with incorrect responses as well as a lower response threshold. According to the response conflict theory modified by Yeung, Botvinick, and Cohen^14^, lower N2b amplitudes reflect fewer conflict signals (N2b amplitude $\propto \mathrm{correct activation }\times \mathrm{incorrect activation}$, Fig. 6A,B) arising from the ACC (Fig. 6C,D). Because in the current study accuracy did not change after the exercise intervention, the improvement in choice reaction times may be due to a decrease in incorrect activations (noise), which led to the reduction of conflict, which, in turn, was likely due to changes in neuromodulators such as catecholamines in the CNS^74^. With smaller incorrect activations (Fig. 6B) leading to a lower response threshold, it would have been possible to improve speed without sacrificing accuracy (Fig. 6).

The finding that the predicted change in inhibitory control was not observed might be ascribed to excessive physiological and/or psychological stress. Although performing five repetitions at 75% of “absolute intensity” (1RM) could be considered as only moderately heavy relative intensity (87%)^75^, the resulting physiological stress might still have been too high in some individuals for optimal inhibitory control given that participants were physically active but untrained adults^76^ (relative intensity is calculated as $(\mathrm{intensity\;performed})\div(\mathrm{maximal\;intensity\;a\;participant\;can\;complete\;for\;a\;given\;number\;of\;repetitions})$^75^; in this case, the intensity performed was 75% 1RM and the predicted 5RM was 86%^51^, thus $75\div 86 = 87$). This result may not be generalizable to other populations such as sedentary, young, or resistance-trained participants, and adjustment of the training parameters will be required to produce optimal improvements in inhibitory control and information processing speed.

### Strengths and limitations

Strengths of this study: (1) applying a crossover RCT design (2) applying multiple joint, free weight, structural exercises, which was intended to improve muscle strength, related connective tissues, and daily function as an intervention (3) applying an active-control physical test and intervention (4) applying a familiarization session before the strength test (5) control of circadian rhythms (6) collecting ERP data (7) conducting a strict intention-to-treat analysis of the behavioral data.

Limitations of this study: (1) Despite the beneficial effect of acute RE on information processing speed being greater than 1 SEM, this effect size failed to reach the more conservative criteria of 2 SEM and 2.77 SEM^70^. (2) Although daily circadian rhythms were controlled, the intervals between sessions weren’t controlled strictly (average ± SD between visits = 7.9 ± 3.2 days). (3) The cognitive task was conducted only 10–35 min after the intervention and it is not clear how long the effect continues beyond this time. (4) Optimization of strength gain cannot be achieved by using only moderate intensity such as 75% of 1RM. Thus, whether RE protocol with higher intensity can produce a similar beneficial effect on information processing speed required further investigation.

The current study was an advancement over previous work in that one individual performed all testing, allocation, and training sessions with all participants and thus avoided inter-rater and inter-instructor variability. This same feature however also lowered the ecological validity of the study and made the blinding of exercise instructors impossible. In addition, the fact that the current study failed to control the diet on or between visit days increased the ecological validity but did so at the expanse of internal validity.

### Practical application

Arranging a single bout of multiple joint, structural barbell resistance exercise (back squat, press and deadlift) with 75% 1RM × 5 repetitions × 3 sets with 2–3 min rest between sets and exercises 10 min before a cognitively demanding task, appears to benefit information processing speed.

### Suggestions for future research

1. Controlling or restricting the time interval between sessions—we recommend that this be 48–96 h, long enough to allow complete recovery, but not so long that participants forget the exercises introduced in previous sessions
2. To allow for greater ability to generalize to other populations, future studies could explore the effect of RE in samples of a) females b) young adults c) resistance-trained participants because of the potential effects of sex hormones, age, and training status on responses to RE.
3. A previous study^23^ has reported that the effects of acute RE on cognition disappeared 180 min after the intervention, but the question of whether the effect persists 35–180 min after exercise remained unexplored.
4. Finally, to enhance the ability to compare studies, it is important that future research report a) descriptions of exercise movements b) the absolute, relative, and allometric strength of the participants c) number of repetitions d) number of sets e) objective and subjective intensities f) length of rest intervals between sets and exercises g) speed of the concentric and eccentric phases of movements h) who (together with their expertise, background) conducts the physical tests and provides the exercise instructions^77^.

## Conclusion

This trial indicated that (1) this type of acute RE intervention improves information processing speed, which may be associated with a decrease in conflict-related neural activity in the ACC (2) the RE protocol used in this trial may have created an inordinate level of stress for improving inhibitory control. Future studies should report resistance exercise parameters in more detail and conduct strength tests only after at least one prior familiarization session. Finally, it is important to employ a standard RCT design and the corresponding analysis to provide more robust results^78^.

## Supplementary Information

Supplementary information.

## Data Availability

The participant-level data and statistical code are available on request from the corresponding author. The data are anonymized and the risk of identification is low although the consent for data sharing was not obtained.
