# Supplementary material for: The effects of barbell resistance exercise on information processing speed and conflict-related ERP in older adults: a crossover randomized controlled trial
Source: Sci Rep. 2021 Apr 28;11:9137. doi: 10.1038/s41598-021-88634-5 (PMC8080630; doi:10.1038/s41598-021-88634-5)
Supplement: Supplementary file 1 — Supplementary information. [file 41598_2021_88634_MOESM1_ESM.pdf]

The Effects of Barbell Resistance Exercise on Information Processing Speed and Conflict-related ERP in Older Adults: A Crossover Randomized Controlled Trial

Authors

Ting-Yu Lin (#1)

Department of Physical Education, National Taiwan Normal University, Taipei, Taiwan (R.O.C.)

leo850922@gmail.com

Shu-Shih Hsieh (#2)

Department of Psychology, Northeastern University, Boston, MA, USA

stonehsieh79218@gmail.com

Ting-Yu Chueh (#3)

Department of Physical Education, National Taiwan Normal University, Taipei, Taiwan (R.O.C.)

gg0229@gmail.com

Chung-Ju Huang (#4)

Graduate Institute of Sport Pedagogy, University of Taipei, Taipei, Taiwan (R.O.C.)

crhwang@utaipei.edu.tw

Tsung-Min Hung (#5*, Corresponding Author)

Department of Physical Education, National Taiwan Normal University, Taipei, Taiwan (R.O.C.)

Institute for Research Excellence in Learning Science, National Taiwan Normal University, Taipei, Taiwan (R.O.C.)

ernesthungkimo@yahoo.com.tw

Supplementary Table S1

Demographic, Anthropometric measures and Physical test

	Intervention sequences Mean (SD)		
	RE to SE (n = 10)	SE to RE (n = 14)	Total (n = 24)
Age	59.3 (4.1)	59.9 (4.1)	59.6 (4.0)
Height (cm)	170.0 (4.5)	167.8 (7.5)	169.0 (6.5)
Weight (kg)	71.9 (7.0)	66.6 (7.4)	68.8 (7.5)
BMI (kg/m^2)	24.7 (2.2)	23.6 (1.9)	24.0 (2.1)
Educational years	15.9 (1.9)	16.2 (1.6)	16.1 (1.7)
Economic status	4.7 (0.7)	4.4 (0.8)	4.5 (0.7)
MMSE	28.8 (0.9)	28.4 (1.3)	28.6 (1.1)
BDI	1.0 (1.9)	1.5 (2.8)	1.3 (2.4)
Physical activity			
Total Activity (min/wk)	172 (72)	161 (87)	165 (80)
Vigorous (MET/wk)	480 (880)	529 (907)	520 (877)
Moderate (MET/wk)	1772 (1262)	841 (752)	1229 (1078)
Walk (MET/wk)	1289 (1069)	1299 (752)	1295 (789)
Total (MET/wk)	3541 (1524)	2689 (1556)	3044 (1569)
Flexibility			
Sit-and-reach (cm)	18.0 (14.6)	18.3 (12.0)	18.2 (12.8)
Shoulder mobility (score)	2.4 (0.7)	2.7 (0.3)	2.6 (0.5)
Absolute strength (kg)			
Squat	63.5 (16.0)	59.5 (10.7)	61.2 (13.0)
Press	33.5 (4.5)	31.6 (4.1)	32.4 (4.3)
Deadlift	75.7 (18.6)	73.3 (9.1)	74.3 (13.6)
Sum	172.8 (36.7)	164.3 (21.3)	167.8 (28.3)
Relative strength (kg/body weight)			
Squat	0.88 (0.24)	0.90 (0.17)	0.89 (0.20)
Press	0.47 (0.05)	0.49 (0.09)	0.48 (0.07)
Deadlift	1.05 (0.29)	1.10 (0.12)	1.08 (0.21)
Sum	2.42 (0.54)	2.48 (0.36)	2.45 (0.42)
Allometric strength (kg/body weight$^{0.66}$)			
Squat	3.79 (0.98)	3.74 (0.67)	3.76 (0.79)
Press	1.99 (0.22)	1.99 (0.25)	1.99 (0.25)
Deadlift	4.52 (1.11)	4.60 (0.50)	4.57 (0.79)
Total	10.32 (2.16)	10.31 (1.27)	10.31 (1.66)

Supplementary Table S2

Rating of Perceived Exertion (RPE) of Resistance Exercises

	Intervention sequence Mean (SD)		
	RE to SE (n = 10)	SE to RE (n = 12)	Total (n = 22)
Squat 1st set	13.3 (1.7)	13.4 (2.4)	13.4 (2.0)
Squat 2nd set	13.2 (1.6)	14.2 (2.2)	13.7 (2.0)
Squat 3rd set	13.9 (1.5)	14.7 (2.5)	14.3 (2.1)
Squat average	13.5 (1.6)	14.1 (2.3)	13.8 (2.0)
Press 1st set	13.4 (1.6)	13.0 (1.5)	13.2 (1.5)
Press 2nd set	13.2 (1.5)	13.7 (1.8)	13.5 (1.6)
Press 3rd set	13.3 (1.2)	14.4 (2.1)	13.9 (1.8)
Press average	13.3 (1.3)	13.7 (1.7)	13.5 (1.5)
Deadlift 1st set	12.9 (1.2)	13.2 (1.5)	13.1 (1.4)
Deadlift 2nd set	13.2 (1.4)	13.8 (1.5)	13.5 (1.5)
Deadlift 3rd set	13.4 (1.4)	14.3 (1.6)	13.9 (1.6)
Deadlift average	13.2 (1.3)	13.7 (1.5)	13.5 (1.4)
Average of all	13.3 (1.2)	13.8 (1.6)	13.6 (1.4)

The scale rates exertion on a scale from 6 to 20 where a rating of 13 corresponds to ‘somewhat hard’ and 15 to ‘hard’.
